# Assessing the long-term implications of age 9 initiation of HPV vaccination on series completion by age 13–15 in the US: projections from an age-structured vaccination model

**DOI:** 10.3389/fped.2024.1393897

**Published:** 2024-06-27

**Authors:** Kunal Saxena, Oscar Patterson-Lomba, Andres Gomez-Lievano, Abigail Zion, Jennifer Cunningham-Erves, Deanna Kepka

**Affiliations:** ^1^ Merck & Co, Rahway, NJ, United States; ^2^ Analysis Group, Inc., Boston, MA, United States; ^3^ Department of Internal Medicine, School of Medicine, Meharry Medical College, Nashville, TN, United States; ^4^ College of Nursing and Huntsman Cancer Institute, University of Utah, Salt Lake City, UT, United States

**Keywords:** adolescent health, vaccine/immunization, public health, HPV, age 9 vaccination

## Abstract

**Introduction:**

Routine human papillomavirus (HPV) vaccination in the US is recommended at ages 11 or 12 years and can be given at age 9. Vaccination completion rates among adolescents 13–15 years in the US remain below the 80% goal. This study evaluated the long-term effects of increasing proactive HPV vaccination initiation rates at age 9 years in completion rates of adolescents.

**Methods:**

An age-structured vaccination model was developed and parametrized based on the National Immunization Survey-Teen (NIS-Teen) survey data. The model projected vaccination coverage (by vaccination status and age group), for 20 years, for a *routine initiation* scenario (no increase in initiation rates of 9-year-olds) and different *proactive initiation* (increased age 9 initiation) scenarios. The time to reach a completion rate of 80% for 13–15-year-olds was estimated. The model also generated projections stratified for subgroups of interest.

**Results:**

Results indicated that vaccine completion rates of 80% in 13–15-year-olds may not be achieved by 2040 under current trends of routine initiation at ages 11 or 12 years. However, increasing initiation rates in 9-year-olds by 1% and 3% annually could shorten the time to achieve 80% completion by 4 and 8 years, respectively. Stratification analyses showed that increasing initiation rates in 9-year-olds can also reduce disparities across subgroups in the time to achieve vaccination completion targets.

**Discussion:**

Increasing HPV vaccination initiation rates in 9-year-olds by as little as 1%–3% annually may be an effective strategy to improve HPV vaccination completion rates in adolescents by age 15 and reach the Healthy People goal of 80% completion much earlier.

## Introduction

Human papillomavirus (HPV) vaccines are a safe and effective way of preventing HPV infection and its related complications (1). However, despite its high safety and effectiveness record, HPV vaccination rates have only marginally increased in recent years and remain below the national target of 80% of eligible 13–15-year-olds completing the vaccine series, a target set by the United States (US) Department of Health and Human Services (Healthy People 2030 goals).^1^ In 2021, only 58.5% of adolescents aged 13–15 years in the US had received the recommended doses of the HPV vaccine, and 50.0–52.9% of adolescents (depending on birth year) completed the series before their 13th birthday (2). In contrast, 89.9% of adolescents in the US have received a dose of tetanus, diphtheria, and pertussis vaccine after age 10, and 86.6% have received the first dose of the quadrivalent meningococcal conjugate vaccine (3). Moreover, HPV vaccination rates among US adolescents were significantly impacted during COVID-19 [e.g., administered doses of HPV vaccines decreased by 24.0% in 2020 compared to 2019 (3)].

In the US, administration of the nona-valent HPV vaccine is recommended by the Advisory Committee on Immunization Practices (ACIP) guidelines for children at 11–12 years of age, with the option to vaccinate children as young as 9 years old at the discretion of a clinician (4). This framing of the guidelines may discourage providers from discussing the vaccine with parents during the 9–10-year-old well-child visits (5). Indeed, the HPV vaccine initiation rate in ages 9 to 10 was 7.5% based on a recent analysis of the National Immunization Survey—Teen (NIS-Teen) data (6). Moreover, in 2018 the American Academy of Pediatrics (AAP) recommended starting HPV vaccination series between ages 9 and 12 years (7).

Lowering the initiation age to 9–10 years can be an effective strategy to increase HPV vaccination rates in the adolescent population. If the first dose in the HPV vaccine series were administered to children at 9–10 years of age, the second vaccine dose could be received before, or concurrently, with other routine vaccinations for children aged 11–12 years. As vaccination uptake rates are high at this age, HPV vaccine coverage is likely to increase as the number of fully vaccinated individuals increases, as recent studies have found. For example, a recent vaccine claims analysis in the US showed that individuals initiating HPV vaccination series at 9–10 years had significantly higher series completion rates by age 13 years compared to those initiating at 11–12 years (8). Similarly, a programmatic intervention study in Denver, Colorado, assessed the impact of changing HPV vaccination initiation from age 11 to 9 years old, finding that the change led to increases in initiation and completion rates by age 13 (9). Using data from NIS-Teen, a recent study also found that initiators at age 9 to 10 were associated with a 27% higher completion rate compared to older initiators (ages 11 and older) (10). A recent survey experiment of clinical staff, who had a role in assessing HPV vaccination status in their practice, suggested that a benefit of recommending vaccine initiation at age 9 compared to age 12 was completing vaccine series by age 13 (11). Results from an intervention study showed that starting vaccination before age 11 resulted in an increase in complete vaccination from 62% to 88% by age 13 (12). Finally, a population-based study in Minnesota, reported that on-time completion of both 2 and 3 doses of the vaccine series by age 13.5 or 15.0 years was significantly associated with initiation at 9–10 years as compared to 11–12 years (13). Studies have also highlighted other benefits of vaccinating adolescents aged 9–10 years. For example, earlier HPV vaccination series completion would increase the chances of protection before sexual debut. Earlier vaccine initiation is particularly important for Black and Latino youths, who are more likely to have an early sexual debut (14), and has been suggested as a strategy for reducing health disparities in HPV-related infections and cancer incidence (15). Evidence from a survey indicates that HPV vaccination before age 11 years showed high parental acceptance owing to reduced stigma relating to sexual activity and the opportunity to administer fewer shots at each visit, and that earlier initiation increased opportunities to complete the series and decreased the need for resource-intensive vaccine recall programs (16). Another survey of primary care physicians showed that over two-thirds of respondents recommend HPV vaccination at ages 9–10 years or are willing to do so (17).

Healthcare organizations have reflected the value of HPV initiation at age 9 in their guidelines. In addition to the AAP recommendations (5), the American Cancer Society (ACS) updated its guidelines stating that “health care providers are encouraged to start offering the HPV vaccine series at age 9 or 10 years” (18). The National Committee on Quality Assurance (NCQA) included completion of HPV series by adolescents' 13th birthday as one of the Healthcare Effectiveness Data and Information Set (HEDIS) measures (19). Despite these guidelines and benefits, the nationwide HPV vaccine initiation rates at ages 9 to 10 remain fairly low relative to initiation at ages 11 or 12 (9, 10, 20).

Motivated by this issue and to complement the existing real-world evidence, in this study we used model simulations to assess the potential impact of increasing rates of earlier initiation of the HPV vaccination series (in 9–10-year-olds) on future vaccination completion rates among adolescents in the US.

## Methods

### Model structure

**Figure 1:**
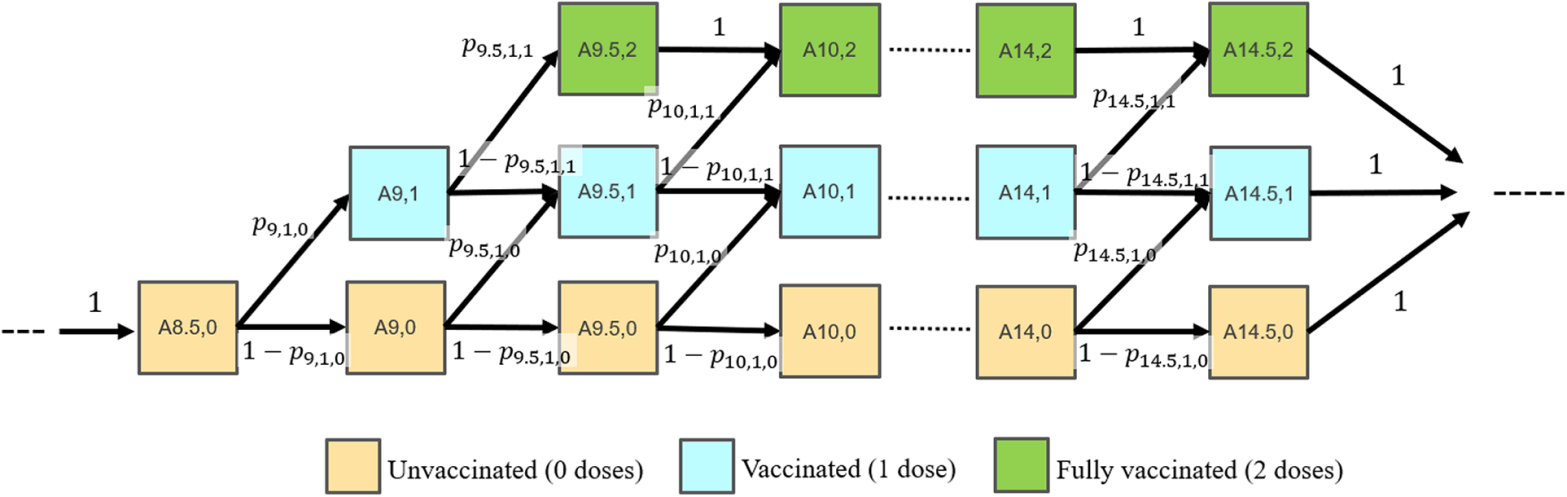
Model diagram. Individuals transition between in 6-month time steps. Model states are labeled as Ai,j for individuals in the (i, j) state, where i represents the age group (8.5, …14.5 years) and j represents the number of total vaccine doses received (0, 1 or 2). Transition probabilities are labeled as pi,j,k, where i represents the age group, j represents the number of vaccine doses received in that age group (0 or 1), and k represents the cumulative number of vaccine doses received in any of the previous age groups (0 or 1).

An age-structured Markov model (21–23) was developed to simulate the effects of increasing HPV vaccine initiation rates in 9–10-year-olds on the vaccination completion rates in 13–15-year-olds. In the model, the proportions of adolescents with different vaccination statuses (unvaccinated; 1-dose; fully vaccinated) within each age group were tracked over time. Those considered “fully vaccinated” in the model (referred to as having received 2 doses) are either those with 2 or 3 provider-confirmed doses who met the ACIP requirements. Individuals transitioned through 6-month age categories (i.e., aged 8.5, 9.0, 9.5, 10.0, … 13.5, 14, and 14.5 years) based on time-varying transition parameters. The upper bound of each age group is not inclusive. For example, 9.5–10-year-olds includes those age 9 and six months up to, but not including age 10, and is denoted by [9.5–10). The 6-month time step duration was selected to reflect the ACIP guidelines^2^ and real-world vaccination patterns in which about 50% of individuals who received 2 doses do so within a 12-month period (6). The model transition probabilities were informed by the NIS-Teen surveys (see Data source section). Within any given time step, individuals could receive 0 or 1 dose. The diagram in Figure 1 shows the model states and transitions.

To reflect in the model outputs that rates of HPV vaccination have, overall, increased in the last decade, the model has a parameter, *b*, which quantifies the baseline, or “natural” increase over time in all the transition probabilities in the model (i.e., capturing systemic increases in vaccination rates in addition to any additional proactive-vaccination intervention). A value of *b = 0* implies no “natural” increase in vaccination rates, whereas a value of *b > 0* leads to increases in vaccination rates. In the simulations, it was assumed that *b = 0.005* [aligned with the data in the 2021 NIS report^3^] starting in 2023 (the start of the simulations) to model the natural increase in vaccination rates in the absence of any proactive-vaccination intervention.

Additional details are provided in Supplementary Materials A regarding the model input parameters, the model transition matrix, and the initial conditions.

### Data source

Model transition probabilities (see Supplementary Table S1) were estimated using the 2022 and 2021 NIS-Teen surveys, which were the most recent data available at the time of this assessment. The NIS-Teen survey seeks to generate timely estimates of immunization coverage rates for all teenage vaccines recommended by the ACIP (24). See Supplementary Materials B for more details on how these data were used to derive the model transition probabilities, along with additional details about assumptions, calculations, and estimates. Data were de-identified and comply with the patient requirements of the Health Insurance Portability and Accountability Act (HIPAA) of 1996; therefore, no review by an institutional review board was required.

Because this analysis studied vaccination of 9–14-year-olds and NIS-Teen 2022 data surveyed 13–17-year-olds, the most recent data on vaccination at age 9 can be provided by 13-year-old survey respondents in 2022. Vaccination rates of 13, 14, 15, 16, and 17-year-olds reported in 2022 correspond to vaccines received by 9, 10, 11, 12, and 13-year-olds in 2018, respectively. Because the span of 9–14-year-olds is one year greater than the data available in a single year of survey data of 13–17-year-olds, vaccination rates for 14-year-olds in 2018 are provided by data of 17-year-olds in 2021. For the main analysis, it was assumed that the transition probabilities in 2022 were the same as in 2018. The rationale behind this assumption is that, although during the 2018–2019 period the vaccination rates increased, they decreased during the 2019–2022 period (due to COVID-19) by a similar amount (3, 25).

### Vaccination scenarios

The model simulated two scenarios:

• **Routine initiation (*status quo*)**. The model projections are entirely based on current empirical data from NIS-Teen for all age groups, with no proactive initiation in place and the majority of initiating happening at ages 11 and 12. The transition probabilities for all age groups are assumed to increase based on current historical trends (i.e., by a baseline amount *b = 0.005* each year).
• **Proactive initiation**: In this scenario, it is assumed that the percentages of individuals who initiate the HPV vaccination series in the age groups 9- and 9.5-year-olds increase linearly over time. Specifically, the probabilities of those in age groups 9- and 9.5-year-olds receiving the first dose (*p_9,1,0_* and *p_9.5,1,0,_* respectively) are increased linearly (i.e., by 3% each year for 20 years), starting from the values determined by the data. This 3% is in addition to the historical baseline increase (i.e., a total of 3.5% yearly increase in initiation probabilities). The transition probabilities for the older age groups (10 years and older) are based on empirical data from NIS-Teen and are assumed to increase based on current historical trends, as in the routine initiation scenario.

### Model outputs

The model generated 20-year projections, starting in 2023, of vaccination coverage for the two scenarios above over time for each age group. The time to reach a 2-dose vaccination completion rate of 80% was also estimated for 13–15-year-olds [the cohort of interest for HPV vaccination goals in the US as stated by US Health and Human Services,^1^ American Cancer Society,^4^ and the National Cancer Institute^5^], as a function of different annual increases (from 1% to 10%) in the initiation rates of 9- and 9.5-year-olds. Since the population sizes of each age group in the model are the same, the completion rate in the group of adolescents aged [13–15) years old was calculated as the average completion rate of the age groups 13–13.5, 13.5–14, 14–14.5 and 14.5–15-year-olds.

### Stratifications

The model also generated projections within subgroups available in the 2021 and 2022 NIS-Teen data, and for which differences in HPV vaccination rates were previously identified (26, 27), including: race and ethnicity [non-Hispanic (NH) White, NH Black, NH other/multiple, and Hispanic]; sex (male and female); income (below poverty, above poverty and below $75,000, over $75,000); census region (Northeast, Midwest, South, and West); and facility (all public facilities, all hospital facilities, all private facilities, all others, mixed, and unknown).

### Sensitivity analysis

In the main analysis, we focused on results based on a proactive initiation intervention characterized by a 3% annual increase in initiation rates for 9- and 9.5-year-olds. To better assess the impact of different proactive initiation interventions, in the sensitivity analysis it was instead assumed that annual increases in initiation rates for those in age groups 9- and 9.5-year-olds would be 1% (pessimistic scenario) and 5% (optimistic scenario), in addition to the historical baseline increase of 0.5%.

The model was simulated in R (28).

## Results

### Vaccination coverage projections

The initial distribution (in 2023) of vaccine initiation and completion rates by age group is in Supplementary Figure S2 in Supplementary Materials C. As expected, initiation rates at age 9 are relatively low compared to other age groups, and initiation rates increase gradually with age. Specifically, initiation rates (percentage of those with 1 or 2 doses) were 2.0% for the 9-year-olds, whereas initiation rates ranged from 31.0% to 62.6% for [11–11.5) and [12.5–13) year-olds, respectively. Initiation rates were highest (76.4%) for the age group of [14.5–15) year-olds.

**Figure 2:**
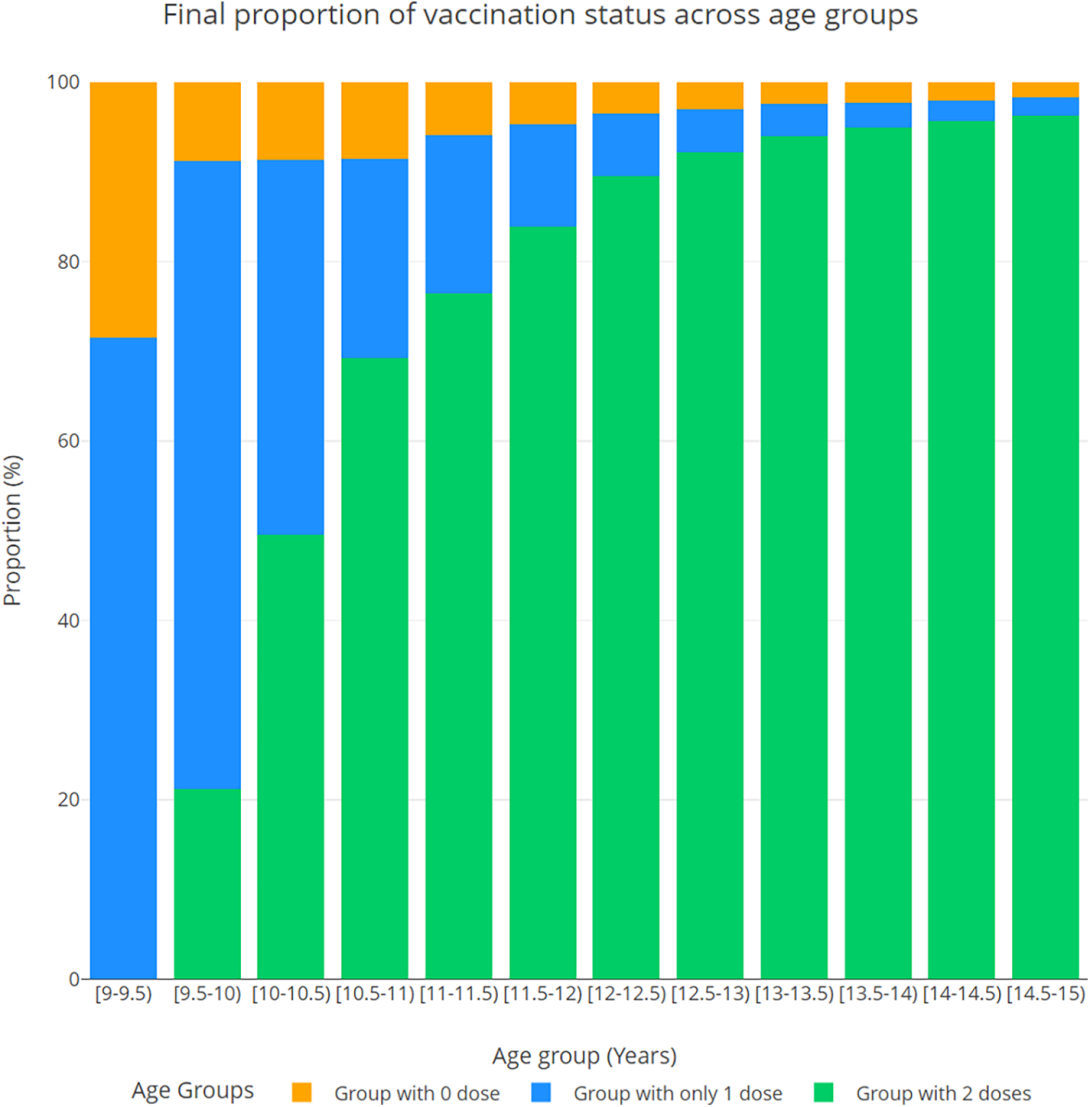
Distribution of vaccine initiation and completion (the percentage of those with 0, 1, and 2 doses) projected in 2043, by age group, in the proactive initiation scenario.

Figure 2 shows the distribution in 2043 of vaccine initiation and completion rates by age group, in the proactive initiation scenario. By year 2043, the initiation rate (the percentage of those with 1 or 2 doses) for 9-year-olds increased to 81.4%, on average, while the initiation rate for 14-year-olds reached 98.1%.

**Figure 3:**
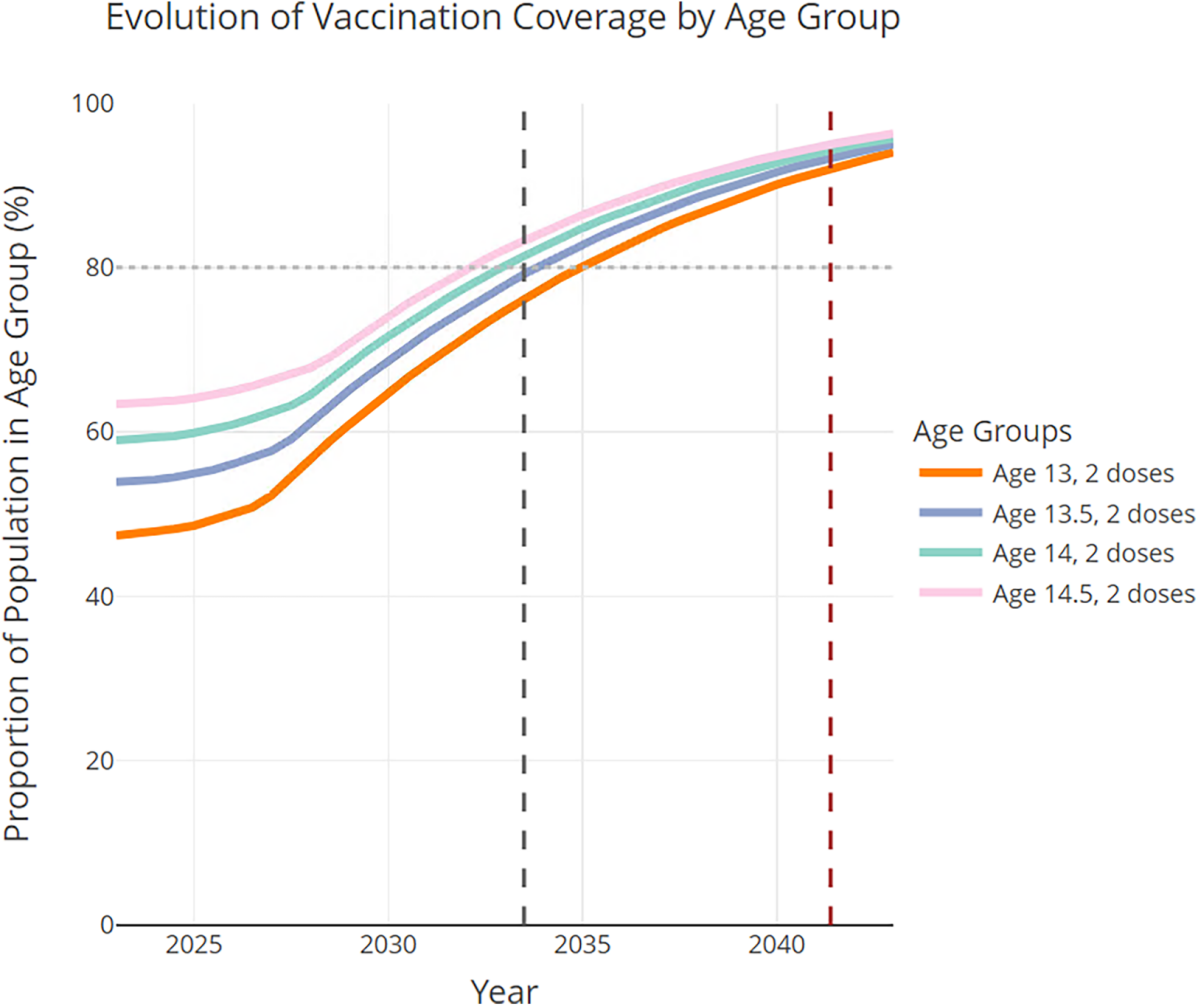
Proactive initiation scenario projections in the 13–15 age groups, assuming all transition probabilities increase according to the historical trends combined with the proactive-initiation strategy (i.e., initiation rates increase by 3.5% each year in age groups 9- and 9.5-year-olds after 2023). The dashed black vertical line indicates the time at which the 80% completion rate is achieved in [13–15) year-olds. The time to reach the 80% completion rate for the routine initiation scenario is marked by the red dashed vertical line.

Figure 3 shows the corresponding proactive initiation scenario projections for adolescents [13–15) years of age. The desired 2-dose vaccine completion (80%, horizontal dashed line) in [13–15) year-olds was achieved between 2033 and 2034, that is, in 10.5 years from 2023, or in 8 years sooner than the routine initiation scenario (see black dashed vertical line). For reference, under the routine initiation scenario, the desired 2-dose vaccine completion in 13–15-year-olds was achieved between 2041 and 2042 (i.e., 18.4 years after 2023, see red dashed vertical line in Figure 3).

### Time to reach 80% vaccination completion rate

**Figure 4:**
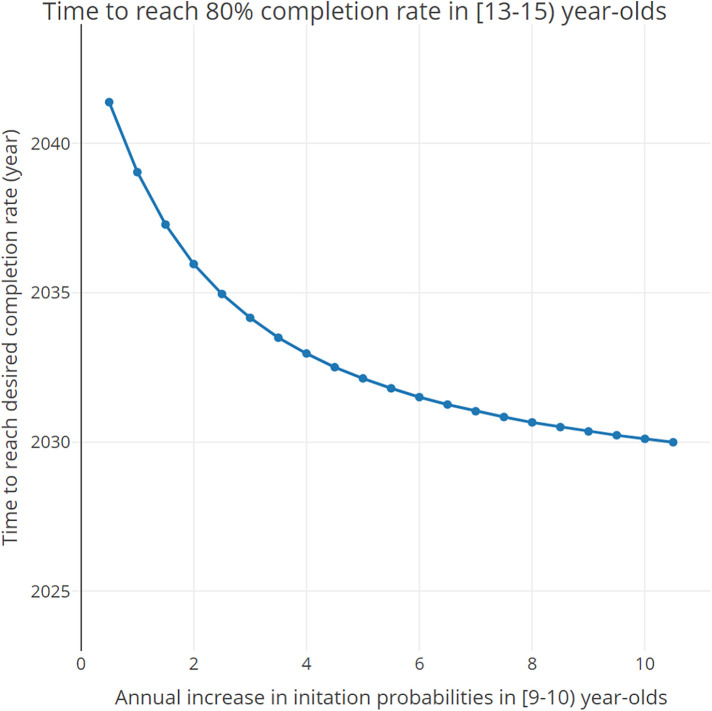
Time needed to reach the desired 80% completion rate in [13–15) year-olds as a function of the total annual increase (baseline annual increase + additional increase) in the initiation probabilities in 9- and 9.5-year-olds.

Figure 4 shows how the time to reach the desired completion rate in [13–15) year-olds changes as a function of the annual increase in the initiation probabilities in 9-year-olds. Results indicate that, if an intervention is put in place to increase the initiation rates of 9-year-olds by 1% each year (1.5% total annual increase), the desired completion rates of 80% in [13–15) year-olds will be achieved between 2037 and 2038. If said intervention were to increase the initiation rates for 9-year-olds by 10% each year (10.5% total annual increase), the desired completion rate will be achieved between 2029 and 2030.

### Sensitivity analysis

In the 1% proactive initiation scenario, the 80% completion rate in [13–15) year-olds would be achieved between 2037 and 2038. In the 5% proactive initiation scenario, it would be achieved by 2032. See Supplementary Materials D for more details.

Table 1 summarizes the model-predicted times to reach 80% vaccination completion in [13–15) year-olds across the core and sensitivity analyses, compared by proactive initiation rates (1%, 3%, 5% and 10%). The results reveal the non-linear relationship between the proactive initiation rate and the time to reach the 80% vaccination completion rate.

**Table 1:** Reduction in time to reach 80% 2-dose vaccination completion in [13–15) year-olds, compared by proactive initiation rate.

Proactive initiation rate^a^ in 9-year-olds	Time to achieve 80% vaccination completion in [13–15) year-olds	Reduction in time to 80% completion, compared to *status quo*
0% (*status quo*)	2042	Reference
1%	2038	4 years
3%	2034	8 years
5%	2032	10 years
10%	2030	12 years

^a^ This rate does not include the baseline rate *b*. This rate represents the magnitude of the different proactive initiation interventions.

### Stratification analysis

**Figure 5:**
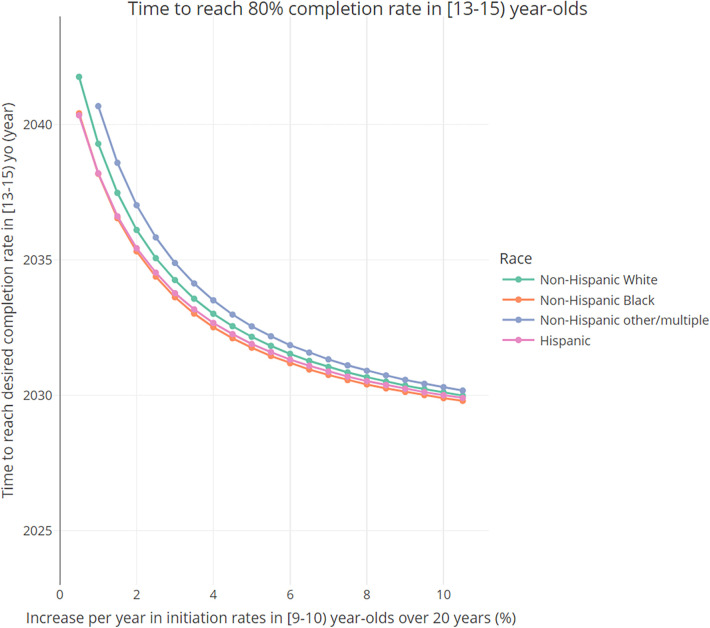
Time needed to reach desired 80% completion rate in [13–15) year-olds, as a function of the total annual increase (baseline annual increase + additional increase) in the initiation probabilities in 9- and 9.5-year-olds, by race and ethnicity.

In general, completion rates are higher among Hispanic and NH Black groups (see Supplementary Figure S7, which shows the initial distribution in 2023 of vaccine completion rates by age group and compared by race and ethnicity). Figure 5 shows how the time to reach a desired completion rate in [13–15) year-olds changes, by race/ethnicity, as a function of the annual increase in the initiation probabilities in 9-year-olds. As the initiation probabilities in 9-year-olds increase, the time to reach the desired completion rate decreases across all racial groups, with Hispanic and NH Black groups reaching it faster than other groups. Notably, for larger values of the initiation rate increase per year, the differences between racial groups become less pronounced.

Supplementary Materials E contains analogous results stratifying by sex, income, region, and facility. In summary, results indicate that, initially, completion rates are similar between sexes (but higher among females), highest among those living below poverty, highest among those in the Northeast region and lowest for those in the South region, and highest for those that get vaccinated in private facilities and lowest in sexually transmitted disease (STD)/school/teen clinics. Similar to Figure 5, as the yearly increase in initiation rates gets larger, differences between strata become less pronounced.

## Discussion

HPV vaccination rates continue to remain below national goals of 80% of eligible 13–15-year-olds completing the vaccine series, and lag behind other routinely recommended vaccinations for adolescents. We posited that increasing HPV vaccine initiation at age 9–10 years could more quickly increase completion rates to and beyond the national goals. Indeed, the positive relationship between early start of vaccination and increases in completion rates among adolescents is validated by several real-world studies (8, 9, 11, 13). However, despite the real-world evidence highlighting the benefits of proactive HPV vaccine, HPV vaccination initiation rates in 9–10-year-olds remain low.

To complement the existing real-world evidence on the impact of proactive HPV vaccination, this study generated long-term modeling evidence to assess, quantitively, the projected effects of increasing rates of initiation of the HPV vaccination series in 9-year-olds on the vaccination completion rates among older adolescents. Projections indicate that desired levels of vaccine completion coverage of 80% in 13–15-year-olds may not be achievable by 2040 under current historical trends, unless interventions are put in place to accelerate initiation rates (e.g., stronger ACIP recommendation to initiate HPV vaccination at age 9).

This model serves as a proof of concept that an effective solution to shorten the time to achieve a completion coverage of 80% in 13–15-year-olds is to substantially increase the HPV vaccine series initiation rates in 9-year-olds. Even small increases in vaccination rates of 9-year-olds can have a substantial impact on reducing the time to achieve the completion coverage goals. Moreover, the results indicated that there is a non-linear relationship between the time to reach a desired completion rate in 13–15-year-olds and the annual increase in the initiation rates in 9-year-olds: initial increments in the proactive initiation rates have more impact on reducing the time to reach 80% completion, and additional increments have relatively lower impact (i.e., a diminishing returns profile). An encouraging interpretation of this observation is that, even proactive initiation interventions that lead to small incremental improvements in vaccination rates of 9-year-olds can have a relatively significant impact on reducing the time to reach the 80% completion goal.

Projections in subgroups of interest revealed differences in initial vaccine completion rates and time to reach desired completion, in line with previous studies.^5^ While subgroups across demographic and economic risk factors converge in terms of time to reach desired completion at higher increases in HPV vaccination initiation rates in 9-year-olds, substantial differences were found between subgroups (e.g., Hispanic vs. non-Hispanic groups) at lower increases in initiation rates. Interestingly, these analyses suggested that an intervention that increases considerably the initiation rates in 9-year-olds has two simultaneous beneficial effects: (1) it reduces the time to reach the desired completion rates for the entire population, and (2) it reduces the differences across groups in the time to achieve the desired completion goals (e.g., interventions that substantially increase initiation rates in 9-year-olds can disproportionately benefit racial/ethnic groups with lower initial vaccination rates). The second observation is aligned with two previous real-world findings. One study suggested that completion rates were higher, and similar across sex and insurance status, among proactive initiators (ages 9 to 10), but were significantly different among older initiators (ages 11 and older) (10). The other one suggested HPV vaccination initiation at age 9 as a strategy for reducing health disparities in HPV-related infections and cancer incidence (17). These findings also suggest that proactive HPV vaccination would not only shorten the time to reach vaccination completion goals, but should also lead to reaching elimination goals faster, not just for cervical diseases but all HPV-related diseases in a gender neutral manner.

An important methodological contribution of this study was a novel approach to estimate age-group specific transition probabilities based on NIS-Teen retrospective survey data. Based on this approach, we were able to not only parameterize the model, but also derive the empirical distribution, across age groups, of vaccination initiation and completion rates for a particular year, thus, broadening the usefulness of the NIS-Teen data.

The predictions presented herein should be interpreted considering important model assumptions. The Markov assumption, which states that the transitions between states are independent of past transitions, implies that the administration age of the second HPV vaccine dose is independent of the age of first dose. However, in the real world, proactive initiators may tend to complete HPV vaccine series earlier than the Markov assumption would predict (e.g., proactive initiators tend to be individuals with better healthcare access and with providers using effective reminders for vaccinating their patients). The Markov assumption, however, is justified not only because it made the model tractable, but also because it arguably generates conservative estimates, in the sense it will not underestimate the time to reach desired completion levels. That is, if increasing the probability of getting the first dose of the vaccine also increases the probability of getting the second dose, then completion goals will be met even earlier than those we projected here.

Our model thus assumes an intervention that only affects the initiation rates of 9-year-olds, and that the overall behavior of individuals remains unchanged otherwise. See Supplementary Materials A for additional discussion on model assumptions. Additionally, the assumption of a constant baseline increase (*b *= 0.5%) over time in all the transition probabilities in the model may be optimistic, particularly for the age 9–10 age groups, which in turn may lead to an underestimation of the time to reach the desired completion goals under the routine initiation scenario. Similarly, we assumed a linear increase in age 9 initiation rates over a 20-year period, whereas historical data suggests that the rates could plateau as rates gets higher. The feasibility of a continuous increase in age 9 vaccination rates will partly depend upon stronger healthcare provider recommendations to curtail parental hesitancy to vaccinating their children at age 9. This hesitancy typically stems from a myriad of reasons including lack of awareness, believing the vaccine is not necessary for their children (e.g., who are not sexually active yet, or are male), or perceived safety concerns [despite robust long term evidence of HPV vaccine safety^6^] (29, 30). That said, there is substantial evidence that HPV vaccination at 9 could help ameliorate some of these issues (see discussion above). Our model does not explicitly take into account the potential effects of vaccine hesitancy on vaccination uptake; however, we do try to account for scenarios of lower vaccination uptake (due in part to hesitancy) by modeling a “pessimistic scenario” with small increases in initiation rates over a long period of time.

Also, when interpreting the results of our model, it should be noted that while an increase in initiation rates at age 9 may lead to less people being vaccinated at a given older age group (e.g., if more people receive their first dose at age 9, then there will be less people eligible to receive their first dose at age 11, even if vaccination rates remain the same for that age group), it does lead to an increase in the total number of people vaccinated by or at a given older age group (e.g., the total number of people who received their first dose by or at age 11 would be larger). Finally, due to data limitations, not all subgroups of interest could be studied in this analysis. For example, assessing differences in vaccination rates between children with and without underlying conditions, or children living in rural vs. urban areas could contribute valuable insights for targeted HPV vaccination campaigns.

In conclusion, the model developed in this study indicates that proactive HPV vaccination, specifically in 9-year-olds, may enable the US to reach the desired 80% HPV vaccination completion goals more swiftly than if age at vaccination stays at 11–12 years. These findings can inform interventions to improve HPV vaccination series completion.

## Data Availability

Publicly available datasets were analyzed in this study. This data can be found here: https://www.cdc.gov/vaccines/imz-managers/nis/datasets-teen.html.

## Footnotes

^1^Available online at: https://health.gov/healthypeople/objectives-and-data/browseobjectives/vaccination/increase-proportion-adolescents-who-get-recommended-doseshpv-vaccine-iid-08 (Accessed September 15, 2023).

^2^Available online at: https://www.cdc.gov/vaccines/vpd/hpv/hcp/recommendations.html#:~:text=HPV%20vaccine%20is%20recommended%20for,not%20adequately%20vaccinated%20when%20younger (Accessed September 15, 2023).

^3^Available online at: https://www.cdc.gov/mmwr/volumes/71/wr/mm7135a1.htm#T1_down (Accessed September 15, 2023).

^4^Available online at: https://www.cancer.org/health-care-professionals/hpvvaccination-information-for-health-professionals/our-hpv-vaccination-initatives.html (Accessed September 15, 2023).

^5^Available online at: https://progressreport.cancer.gov/prevention/hpv_immunization (Accessed September 15, 2023).

^6^Available online at: https://www.cancer.gov/news-events/cancer-currents-blog/2021/hpv-vaccine-parents-safety-concerns (Accessed September 15, 2023).
