# Supplementary material for: Assessing the long-term implications of age 9 initiation of HPV vaccination on series completion by age 13–15 in the US: projections from an age-structured vaccination model
Source: Front Pediatr. 2024 Jun 27;12:1393897. doi: 10.3389/fped.2024.1393897 (PMC11238570; doi:10.3389/fped.2024.1393897)
Supplement: Supplementary file 1 [file Datasheet1.docx]

# *Supplemental Materials*

# Assessing the Long-Term Implications of Age 9 Initiation of HPV Vaccination on Series Completion by Age 13-15 in the US: Projections from an Age-Structured Vaccination Model

# Supplemental Materials A: Additional model details

## Model structure

Within any given time step, individuals can receive 0 or 1 dose, but not 2 doses. This model assumption is aligned with the recommendation of receiving a second dose of HPV vaccine 6-12 months after the first one. The diagram in Figure S1 shows the model states and transitions. Model states are labeled as *A_i,j_* for individuals in the (*i, j*) state, where *i* represents the age group (9, 9.5, …14.5 years) of the adolescent and *j* represents the number of total vaccine doses received (0, 1 or 2). For example, “A9.5, 1” are those in the 9.5-year-olds group (i.e., age 9 and six months to 10 [not inclusive for the upper bound]) who have received 1 total HPV vaccination dose up to that age. The fraction of individuals with 0, 1 or 2 doses (i.e., fully vaccinated) in each age group represents, at any given time, the coverage rates (that is, the vaccination status: 0-doses vs 1-dose vs 2-doses [fully vaccinated]) in that age group.

Model state transition probabilities are labeled as *p_i,j,k_*, where *i* represents the age group (9, 9.5, …14.5 years), *j* represents the number of vaccine doses received *in* *that* age group (0 or 1), and *k* represents the cumulative number of vaccine doses received *in any of the* *previous* age groups (0 or 1). For example, *p_10,1,1_* represents the probability of that a 10-year-old had received 1 dose within that age group (i.e., from age 10 to 10 and six months [not inclusive for the upper bound]) and received 1 dose within any of the prior age groups (i.e., from age 9 to 10 [not inclusive for the upper bound]). To simplify the notation, the transitions out of each state are represented by the probabilities of receiving a vaccine in the next age group (*p_i,j,k_*) and its complement (*1-* *p_i,j,k_*).


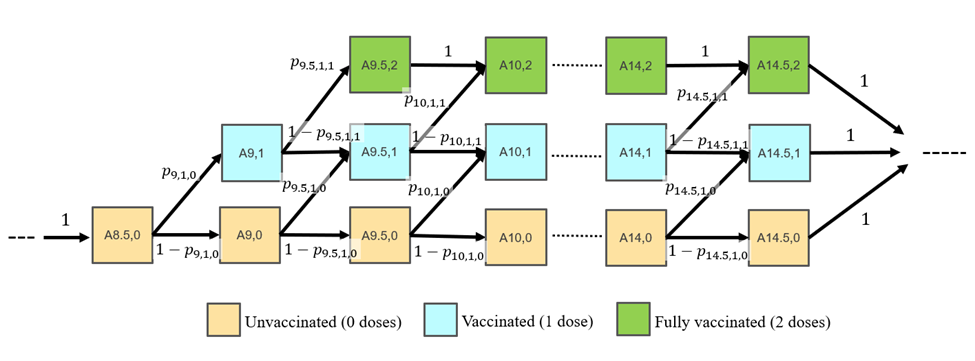


Figure S1: Model diagram. Individuals transition between states following the arrows according to probabilities estimated from real-world data in 6-month time steps. The colors of the model state boxes represent the different vaccination status: yellow for unvaccinated (0 doses), blue for one dose, and green for fully vaccinated (2 doses). The dotted lines in the middle represent the age categories not shown in the figure, for simplicity.

Individuals first transition into the model through the initial state (age group A8.5, or persons aged 8 years and six months to 9 years), and they leave the model after passing through the final states (age group A14.5, or persons aged 14.5 years to 15 years). Thus, the population size through the different age groups is kept constant and normalized to 1 in each age group.

To reflect in the model outputs the fact that rates of HPV vaccination have, overall, increased in the last decade, the model has a parameter, *b*, which quantifies the baseline, or “natural” increase over time in all the transition probabilities in the model (i.e., capturing systemic increases in vaccination rates in addition to any additional proactive-vaccination intervention), such that a value of *b=0* implies no “natural” increase in vaccination rates, whereas a value of *b>0* leads to increases in vaccination rates. The value of *b* was estimated such that *b*=*0.005* (0.5%) is associated with an annual increase of 1-2% in initiation rates for 12-14-year-olds, which is aligned with the empirical changes observed in that age group from 2014 to 2021, based on the 2021 NIS report^[[1]](#endnote-1)^. Thus, in the simulations, it was assumed that *b=0.005* starting in 2023 (the start of the simulations) as to model the expected natural increase in vaccination rates going forward, in the absence of any proactive-vaccination intervention.

A model assumption was setting each age cohort to have the same population size, and have the adolescent population not change with time. Thus, the results from this model are valid for adolescent populations that are not expected to change significantly in the next 10 years.

The annual increase in initiation rates in 9-10 year olds was assumed to be constant across time (e.g., 1%, 3% or 5% per year), as opposed to a more realistic time-varying increase. However, there is a lack of data to support a less parsimonious assumption (e.g., assume that annual increase in initiation rates in 9-10 year olds vary across time) which would also make the model less tractable.

To account for the fact that vaccination rates increased over time, and are expected to continue doing so in the future, it was assumed that all the transition probabilities in the model increased over time by a constant amount *b* each year, which was estimated using historical data. This parameter is expected to be age-group specific, and to vary across time (particularly given the effects of the Covid-19 pandemic). Relaxing these two assumptions would, first, require age- and time-specific vaccination rate data to estimate age- and time-specific values for the *b* parameter, and second, would make the model more complex and thus less tractable; therefore, this study followed a more parsimonious approach, that is expected to provide, on average across all age groups and years, a good approximation of the changes in vaccination rates.

## Model input parameters

The model transition probabilities that are needed as input to the model are listed in Table S1. In the *Data Source* section, a description on how these probabilities were obtained from real-world data is provided.

Table S1. Model parameters

| **Model parameter** | **Parameter label** |  |
| --- | --- | --- |
| Probability of receiving 1 dose at age 9, given no prior doses | *p_9,1,0_* |  |
| Probability of receiving 1 dose at age 9.5, given 1 dose by age 9 | *p_9.5,1,1_* |  |
| Probability of receiving 1 dose at age 9.5, given 0 doses by age 9 | *p_9.5,1,0_* |  |
| Probability of receiving 1 dose at age 10, given 1 dose by age 9.5 | *p_10,1,1_* |  |
| Probability of receiving 1 dose at age 10, given 0 doses by age 9.5 | *p_10,1,0_* |  |
| …. | | |
| Probability of receiving 1 dose at age *n*, given 1 dose by age *n-1* | *p_n,1,1_* |  |
| Probability of receiving 1 dose at age *n*, given 0 doses by age *n-1* | *p_n,1,0_* |  |
| … | | |
| Probability of receiving 1 dose at age 14, given 1 dose by age 13.5 | *p_14,1,1_* |  |
| Probability of receiving 1 dose at age 14, given 0 doses by age 13.5 | *p_14,1,0_* |  |
| Probability of receiving 1 dose at age 14.5, given 1 dose by age 14 | *p_14.5,1,1_* |  |
| Probability of receiving 1 dose at age 14.5, given 0 doses by age 14 | *p_14.5,1,0_* |  |

## Model transition matrix

The evolution of individuals’ vaccination status was determined by the transition matrix, *M*, presented below. At each time step, individuals transitioned to the subsequent age group, and their vaccination status evolved based on the transition probabilities in this matrix. Specifically, the probabilities in each cell (*x, y*) determined the probability of transitioning from the state in column *x* to row *y*. As expected, the sum of probabilities across rows (i.e., for each column) equals 1.

Transition matrix, M, which determines the evolution of individuals’ vaccination status.

| *X* | *A8.5,0* | *A9,1* | *A9,0* | *A9.5,2* | *A9.5,1* | *A9.5,0* | *A10,2* | *A10,1* | *A10,0* | *…* | *A14.5,2* | *A14.5,1* | *A14.5,0* | *y* |
| --- | --- | --- | --- | --- | --- | --- | --- | --- | --- | --- | --- | --- | --- | --- |
|  | *0* | *0* | *0* | *0* | *0* | *0* | *0* | *0* | *0* |  | *1* | *1* | *1* | *A8.5,0* |
|  | *p_9,1,0_* | *0* | *0* | *0* | *0* | *0* | *0* | *0* | *0* | *…* | *0* | *0* | *0* | *A9,1* |
|  | *1-p_9,1,0_* | *0* | *0* | *0* | *0* | *0* | *0* | *0* | *0* | *…* | *0* | *0* | *0* | *A9,0* |
|  | *0* | *p_9.5,1,1_* | *0* | *0* | *0* | *0* | *0* | *0* | *0* | *…* | *0* | *0* | *0* | *A9.5,2* |
|  | *0* | *1- p_9.5,1,1_* | *p_9.5,1,0_* | *0* | *0* | *0* | *0* | *0* | *0* | *…* | *0* | *0* | *0* | *A9.5,1* |
|  | *0* | *0* | *1- p_9.5,1,0_* | *0* | *0* | *0* | *0* | *0* | *0* | *…* | *0* | *0* | *0* | *A9.5,0* |
| *M=* | *0* | *0* | *0* | *1* | *p_10,1,1_* | *0* | *0* | *0* | *0* | *…* | *0* | *0* | *0* | *A10,2* |
|  | *0* | *0* | *0* | *0* | *1- p_10,1,1_* | *p_10,1,0_* | *0* | *0* | *0* | *…* | *0* | *0* | *0* | *A10,1* |
|  | *0* | *0* | *0* | *0* | *0* | *1- p_10,1,0_* | *0* | *0* | *0* | *…* | *0* | *0* | *0* | *A10,0* |
|  | *…* | *….* | *….* | *….* | *….* | *….* | *….* | *…* | *…* | *…* | *…* | *…* | *…* |  |
|  | *0* | *0* | *0* | *0* | *0* | *0* | *0* | *0* | *0* | *…* | *0* | *0* | *0* | *A14.5,2* |
|  | *0* | *0* | *0* | *0* | *0* | *0* | *0* | *0* | *0* | *…* | *0* | *0* | *0* | *A14.5,1* |
|  | *0* | *0* | *0* | *0* | *0* | *0* | *0* | *0* | *0* | *…* | *0* | *0* | *0* | *A14.5,0* |

The evolution of the system is mathematically represented in equation (1).

$$\vec{A}\left( t+1 \right)=M*\vec{A}\left( t \right) \left( 1 \right)$$

where $\vec{A}$ is a 36x1 vector of the 36 model states.

## Initial conditions

The initial conditions for each model state are given by:

*N* = population size of each age group (without loss of generality, *N=1*)

*A8.5,0(t=0) =1*

*A9,1(t=0) =* Prev__9_1_

*A9,0(t=0) = 1 –* Prev__9_1_

*A9.5,1(t=0) =* Prev__9.5_1_

*A9.5,2(t=0) =* Prev__9.5_2_

*A9.5,0(t=0) = 1-* Prev__9.5_1_ – Prev__9.5_2_

*A10,1(t=0) =* Prev__10_1_

*A10,2(t=0) =* Prev__10_2_

*A10,0(t=0) = 1-* Prev__10_1_ – Prev__10_2_

*…*

*A14.5,1(t=0) =* Prev__14.5_1_

*A14.5,2(t=0) =* Prev__14.5_2_

*A14.5,0(t=0) = 1-* Prev__14.5_1_ – Prev__14.5_2_

where Prev__i_j_ represents the initial (or historical) prevalence of individuals in age group *i* that have received *j* vaccine doses. For example, Prev__9_1_ is the prevalence of persons in the 9-year-old age group (age 9 to 9 and six months [not inclusive for the upper bound]) with 1 dose, with denominator being the population size of 9-year-old age group, in the reference period.

The data inputs are the transition probabilities listed in Table S3 and Table S4. The initial conditions, or prevalence values, were required to be consistent with these transition probabilities. Based on linear algebra theory, the steady-state vector *Prev0* of the transition matrix *M* is the vector that satisfies the equation: *M * Prev0 = Prev0*. The eigenvalue of *M* with real part equal to 1 corresponds to the steady state eigenvector *Prev0*. This steady state eigenvector represents, in this model, the initial distribution of prevalence values, up to a constant *C*, of vaccination status across age groups (in other words, it is assumed the system has already reached an equilibrium based on the estimated transition probabilities). Finally, the constant *C* can be determined by using the constraint that the prevalence values need to add up to 1 in each age group.

## Evolution of the model in the Proactive Initiation scenario

In the proactive initiation scenario in the Methods section, the evolution of the system is then mathematically represented by:

$$\vec{A}\left( t+1 \right)=M(t)*\vec{A}\left( t \right)$$

where the transition probability *M* is a function of time due to *p_9,1,0_(t)* and *p_9.5,1,0_(t)*. After *n* time steps, we get:

$$\vec{A}\left( t+n \right)=M\left( t+n-1 \right)*\vec{A}\left( t+n-1 \right)=M\left( t+n-1 \right)*M\left( t+n-2 \right)\ldots*M\left( t \right)*\vec{A}\left( t \right).$$

# Supplemental Materials B: Additional details on the derivations of model inputs

## NIS-Teen data description

The NIS-Teen survey is conducted annually via telephone interviews to parents or guardians of a nationally representative sample of adolescents aged 13-17 years in the United States. Interviews are followed by a mailed questionnaire to adolescents’ immunization providers.^[[2]](#endnote-2)^

With respect to HPV vaccination history, NIS-Teen data provided 4 key variables critical for estimating transition probabilities for our model: (i) the age of the respondent, in years, at the time of the survey; (ii) the number of total doses received by a respondent according to that respondent’s provider; (iii) the age (in months) when adolescents acquired each HPV vaccine dose; and (iv) whether a respondent has received all necessary doses to complete their HPV vaccine schedule. Using these variables, transition probabilities were derived for the model based on when each adolescent reported their first (initiation) and last (series completion) HPV doses.

The goal was to obtain the most recent transition probabilities possible for adolescents from ages 9 years to 15 years (exclusive) of age. However, NIS-Teen data on age at initiation and completion is *retrospective*. Age cohorts 13 years to 18 years (exclusive) in the year 2022 reflect those aged 9 years to 14 years (exclusive) in 2018, which is the most recent year for which vaccination data for 9-year-olds could be obtained, as the youngest survey respondents in 2022 were approximately 9 years old in 2018. Therefore, to obtain data for 14-year-olds to 15-year-olds (exclusive) in 2018, the 2021 data were used, as those respondents had aged out of the survey by 2022 (the exact mapping of cohorts for transition probabilities is detailed in Table S2).

## Retrospective cohort mapping

Table S2 shows 2018 transitions mapped to the age cohort and year they are derived from.

Table S2. Retrospective cohort mapping.

| **Transition in 2018** | **Age cohort, data year** |
| --- | --- |
| [8.5, 9.0) 🡪 [9.0, 9.5) | 13-year-olds, 2022 |
| [9.0, 9.5) 🡪 [9.5, 10.0) | 13-year-olds, 2022 |
| [9.5, 10.0) 🡪 [10.0, 10.5) | 14-year-olds, 2022 |
| [10.0, 10.5) 🡪 [10.5, 11.0) | 14-year-olds, 2022 |
| [10.5, 11.0) 🡪 [11.0, 11.5) | 15-year-olds, 2022 |
| [11.0, 11.5) 🡪 [11.5, 12.0) | 15-year-olds, 2022 |
| [11.5, 12.0) 🡪 [12.0, 12.5) | 16-year-olds, 2022 |
| [12.0, 12.5) 🡪 [12.5, 13.0) | 16-year-olds, 2022 |
| [12.5, 13.0) 🡪 [13.0, 13.5) | 17-year-olds, 2022 |
| [13.0, 13.5) 🡪 [13.5, 14.0) | 17-year-olds, 2022 |
| [13.5, 14.0) 🡪 [14.0, 14.5) | 17-year-olds, 2021 |
| [14.0, 14.5) 🡪 [14.5, 15.0) | 17-year-olds, 2021 |

## Completion transition probability derivation

For explanatory purposes, the respondent cohort for age at vaccination *i* is denoted as *i'*. The numerator for the transition probability of completion entering a given 6-month age group *i* is the total number of respondents from the corresponding age cohort *i’* who received their completion dose of the HPV vaccine in age group *i.* The corresponding denominator is the number of respondents from age cohort *i’* who had initiated but not completed prior to age group *i.*

Because the ACIP updated their dosing recommendations in 2016,^[[3]](#endnote-3)^ the following algorithm was developed for identifying true completion of the HPV vaccine schedule:

- Included in numerator
  - Respondents from age cohort *i’* who had 2 doses at the time of the survey, were indicated as “up-to-date” on ACIP requirements by provider, did not receive their first dose at age *i*,^[[4]](#endnote-4)^ and received their second dose at *i*
  - Respondents from age cohort *i’* who had 3 doses at the time of the survey, were indicated as “up-to-date” on ACIP requirements by provider, did not receive their second dose at age *i*,*^20^* and received their third dose at age *i*
- Included in denominator:
  - Respondents from age cohort *i’* who had 1 dose at the time of the survey and who received their first dose prior to age group *i*
  - Respondents from age cohort *i’* who had 2 doses at the time of the survey, were indicated as “up-to-date” on ACIP requirements by provider, received their first dose prior to age group i, and did not receive their second dose prior to age group *i*
  - Respondents from age cohort *i’* who had 2 doses at the time of the survey, were not indicated as “up-to-date” on ACIP requirements by provider, and received their second dose prior to age *i*
  - Respondents from age cohort *i’* who had 3 doses at the time of the survey, were indicated as “up-to-date” on ACIP requirements by provider, received their second dose prior to age *i*, and did not receive their third dose prior to age *i*

## Assumptions in deriving transition probabilities

The following assumptions were necessary to derive probabilities that describe how, on average, teens received immunizations at different ages.^[[5]](#endnote-5)^

- The model was designed such that in each time step (six months), individuals can receive 0 or 1 dose, but not 2 doses. This model assumption is aligned with the recommendation of receiving a second dose of HPV vaccine 6-12 months after the first one.
- Respondents with any of the following attributes were censored from the derivation of both initiation and completion transition probabilities:
  - Inadequate provider data
  - State of residence in Puerto Rico, US Virgin Islands, or Guam
  - Received first dose of HPV vaccine prior to age 9.0 years (<1% of study sample)
- Respondents with any of the following attributes were censored from the derivation of only completion transition probabilities:
  - Received second or third dose of HPV vaccine prior to age 9.5 years
  - Received more than 3 doses of the HPV vaccine at the time of the survey. Additional doses are in excess to the minimum vaccination completion requirement and may suggest an individual did not wait the recommended 6-12 months between receipt of subsequent doses. Furthermore, additional doses beyond the first and last required for the vaccination schedule are irrelevant to the transition probabilities and vaccination states considered within the model.
  - Received their completion dose in the same 6-month age group as their initiation dose or received their third dose in the same 6-month age group as their second dose. This data is not compatible with model’s necessary assumptions developed to align with ACIP recommendations.
- Indeterminant transition probabilities, or probabilities for which there are no respondents in the numerator nor denominator, were presumed to be 0. Instances of such transition probabilities only occurred for several completion probabilities for stratifications with limited data.

## Transitions from 0 to 1 dose

Using the methodology described above, Table S3 shows the initiation transition probabilities for the overall population in 2018.

Table S3. Transition probabilities from 0 doses to 1 dose for all model age groups.

| Age group (years) | 6-month age group | Model parameter | Transition probability |
| --- | --- | --- | --- |
| 9 | [9.0, 9.5) | $p_{9,1,0}$ | 0.015 |
| 9.5 | [9.5, 10.0) | $p_{9.5,1,0}$ | 0.010 |
| 10 | [10.0, 10.5) | $p_{10,1,0}$ | 0.019 |
| 10.5 | [10.5, 11.0) | $p_{10.5,1,0}$ | 0.021 |
| 11 | [11.0, 11.5) | $p_{11,1,0}$ | 0.263 |
| 11.5 | [11.5, 12.0) | $p_{11.5,1,0}$ | 0.192 |
| 12 | [12.0, 12.5) | $p_{12,1,0}$ | 0.232 |
| 12.5 | [12.5, 13.0) | $p_{12.5,1,0}$ | 0.126 |
| 13 | [13.0, 13.5) | $p_{13,1,0}$ | 0.156 |
| 13.5 | [13.5, 14.0) | $p_{13.5,1,0}$ | 0.064 |
| 14 | [14.0, 14.5) | $p_{14,1,0}$ | 0.125 |
| 14.5 | [14.5, 15.0) | $p_{14.5,1,0}$ | 0.090 |

## Transitions from 1 dose to completion

Using the methodology described above, Table S4 shows the completion transition probabilities for the overall population in 2018.

Table S4. Transition probabilities from 1 dose to completion.

| Age group (years) | 6-month age group | Model parameter | Transition probability |
| --- | --- | --- | --- |
| 9 | [9.0, 9.5) | $p_{9,1,1}$ | 0.000^[[6]](#endnote-6)^ |
| 9.5 | [9.5, 10.0) | $p_{9.5,1,1}$ | 0.201 |
| 10 | [10.0, 10.5) | $p_{10,1,1}$ | 0.315 |
| 10.5 | [10.5, 11.0) | $p_{10.5,1,1}$ | 0.392 |
| 11 | [11.0, 11.5) | $p_{11,1,1}$ | 0.271 |
| 11.5 | [11.5, 12.0) | $p_{11.5,1,1}$ | 0.375 |
| 12 | [12.0, 12.5) | $p_{12,1,1}$ | 0.467 |
| 12.5 | [12.5, 13.0) | $p_{12.5,1,1}$ | 0.357 |
| 13 | [13.0, 13.5) | $p_{13,1,1}$ | 0.374 |
| 13.5 | [13.5, 14.0) | $p_{13.5,1,1}$ | 0.308 |
| 14 | [14.0, 14.5) | $p_{14,1,1}$ | 0.311 |
| 14.5 | [14.5, 15.0) | $p_{14.5,1,1}$ | 0.288 |

# Supplemental Materials C: Initial distribution of vaccination status by age group


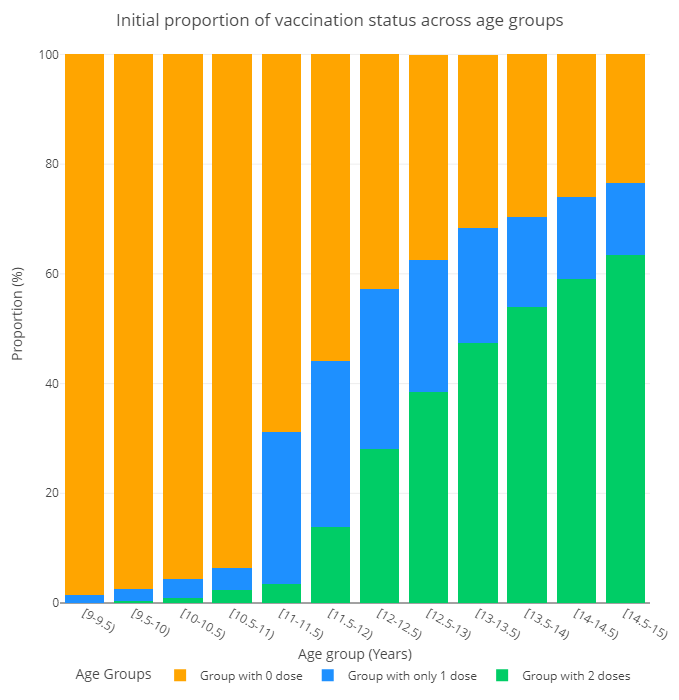


Figure S2: Initial distribution in 2023 of vaccine initiation and completion by age group.

# Supplemental Materials D: Sensitivity analyses

## 1% annual increase in initiation rates (pessimistic scenario)

- Figure S3 shows the *final* distribution (in 2043) of vaccine initiation and completion rates by age group, assuming initiation rates/probabilities increase by 1.5% each year in age groups 9- and 9.5-year-olds after 2023, which includes a 0.5% natural increase and a 1% additional increase.
- Under this proactive-initiation scenario, the initiation rate for the age group of [14.5-15) year-olds reached 95.4%.


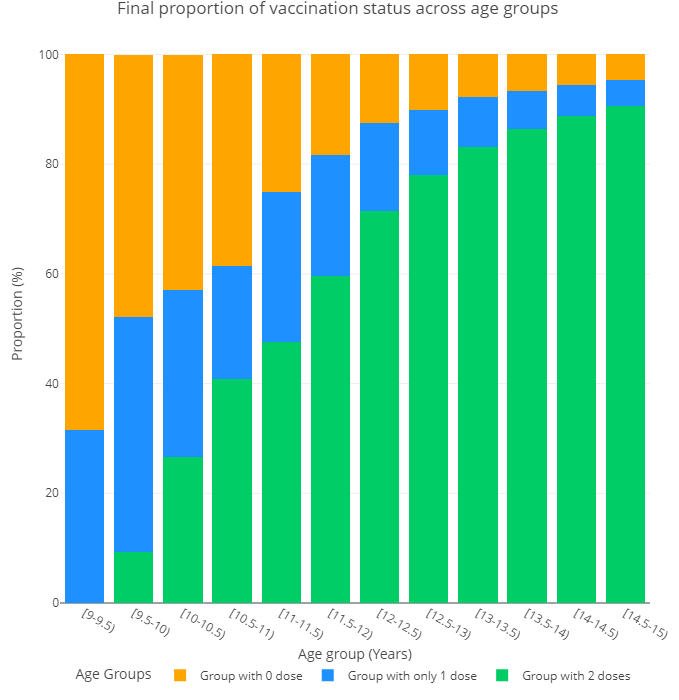


Figure S3: Projected distribution of vaccine initiation and completion in 2043 by age group, with 1% annual increase in initiation rates.

- Figure S4 shows the corresponding proactive-initiation scenario projections for adolescents [13-15) years of age. The desired 2-dose vaccine coverage (80%, horizontal dashed line) in [13-15) year-olds was reached between 2037 and 2038 (in 14.3 years from 2023, see black dashed vertical line). For reference, the time to reach the 80% coverage for the routine initiation scenario is marked by the red dashed vertical line.


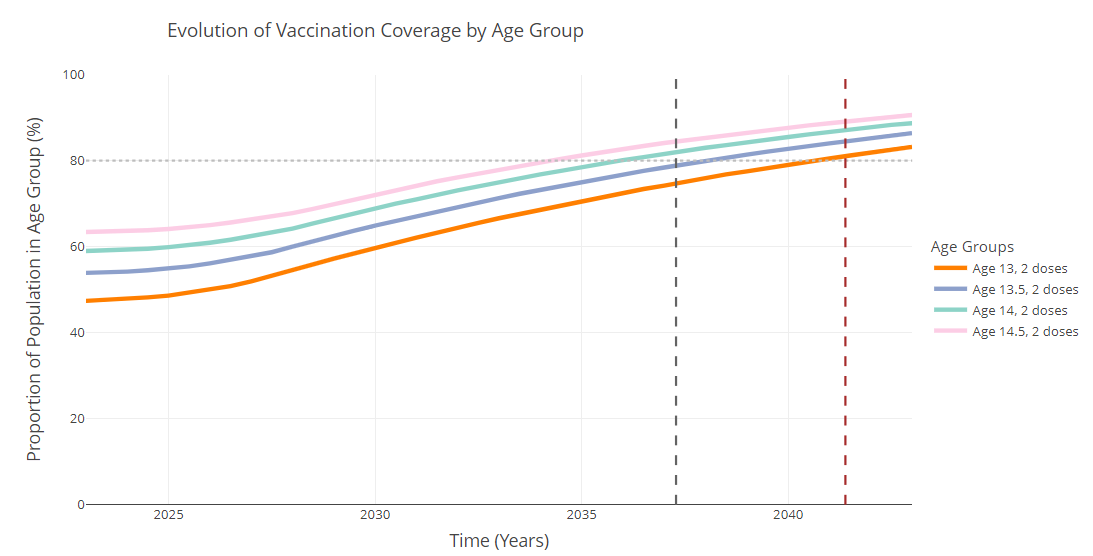


Figure S4: Proactive-initiation scenario projections in the [13-15) age group, assuming all transition probabilities increase according to the historical trends combined with the proactive-initiation strategy (1% annual increase in initiation rates). The desired 2-dose vaccine coverage (80%, horizontal dashed line) in [13-15) year-olds was reached between 2037 and 2038 (see black dashed vertical line). For reference, the time to reach the 80% coverage for the routine initiation scenario is marked by the red dashed vertical line.

## 5% annual increase in initiation rates (optimistic scenario)

- Figure S5 shows the *final* distribution (in 2043) of vaccine initiation and completion rates by age group, assuming initiation rates/probabilities increase by 5.5% each year in age groups 9- and 9.5-year-olds after 2023, which includes a 0.5% natural increase and a 5% additional increase.
- Under this proactive-initiation scenario, the initiation rate for the age group of [14.5-15) year-olds reached 99.8%.


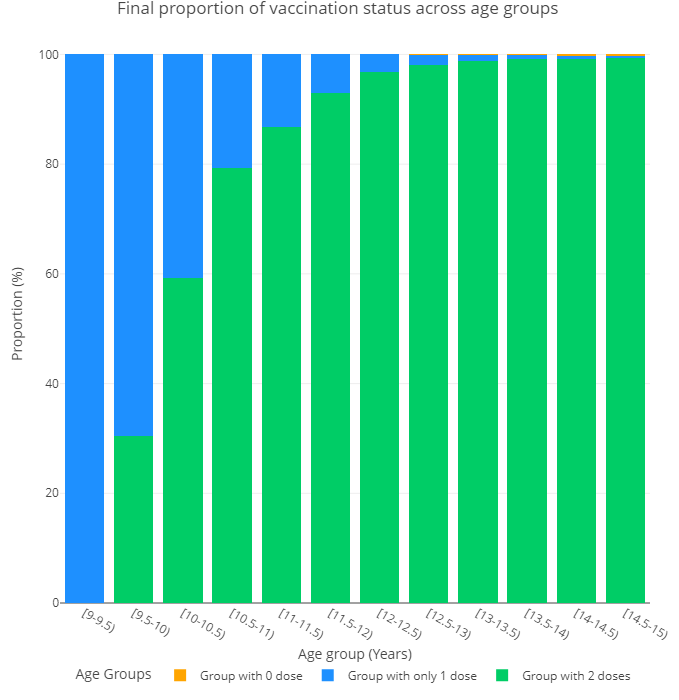


Figure S5: Final (in 2043) distribution of vaccine initiation and completion by age group, with 5% annual increase in initiation rates.

- Figure S6 shows the corresponding proactive-initiation scenario projections for adolescents [13-15) years of age. The desired 2-dose vaccine coverage (80%, horizontal dashed line) in [13-15) year-olds was reached by 2032 (in 8.8 years from 2023, see black vertical dashed line).


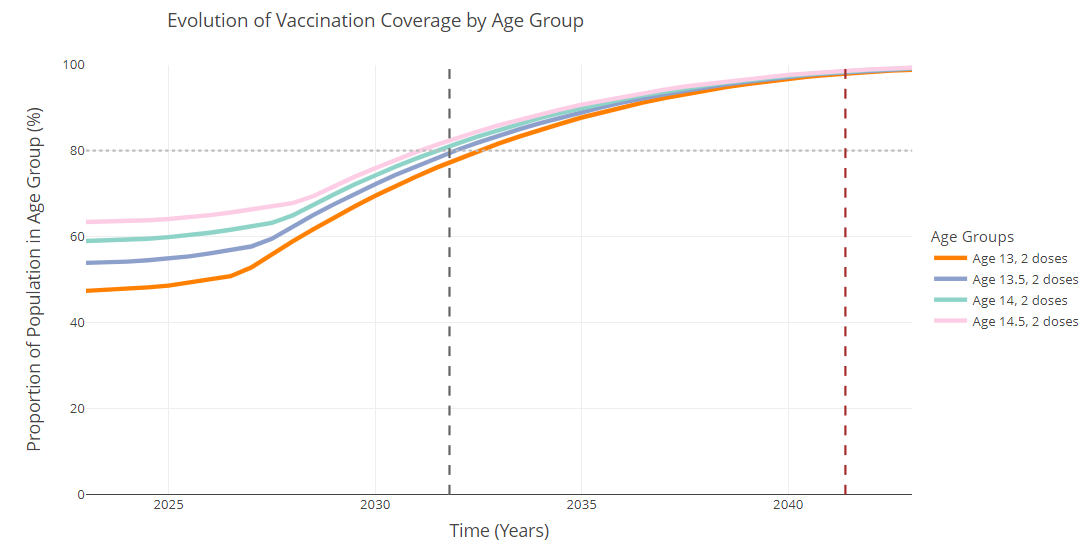


Figure S6: Proactive-initiation scenario projections in the [13-15) age group, assuming all transition probabilities increase according to the historical trends combined with the proactive-initiation strategy (5% annual increase in initiation rates). The desired 2-dose vaccine coverage (80%, horizontal dashed line) in [13-15) year-olds was reached by 2032 (see black dashed vertical line). For reference, the time to reach the 80% coverage for the routine initiation scenario is marked by the red dashed vertical line.

# Supplemental Materials E: Stratification analyses

## Stratification by race and ethnicity


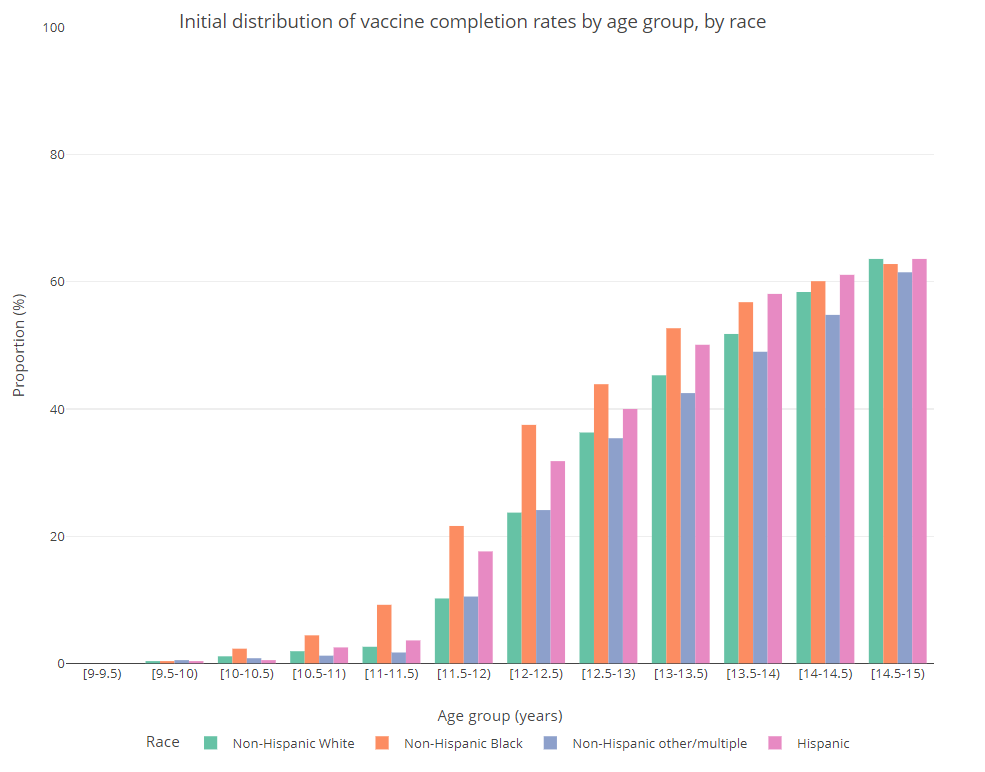


Figure S7: Initial distribution of vaccine completion rates in 2023 by age group, and compared by race and ethnicity

## Stratification by sex

Figure S8 shows the initial distribution of vaccine completion rates in 2023 by age group, and compared by sex (male vs female). The results indicate that, in general, completion rates are similar between sexes, but higher among females. Figure S9 shows how the time to reach a desired completion rate in [13-15) year-olds changes, by sex, as a function of the annual increase in the initiation rates/probabilities in 9-year-olds. For a given increase in proactive initiation rates, the time it takes for females to reach the desired completion rates is shorter (by up to 2 years when the total annual increase in proactive vaccination rates is 1%) compared to males. Similar to the pattern noted for race and ethnicity, as the initiation rate increase per year increases, differences between males and females become less pronounced.


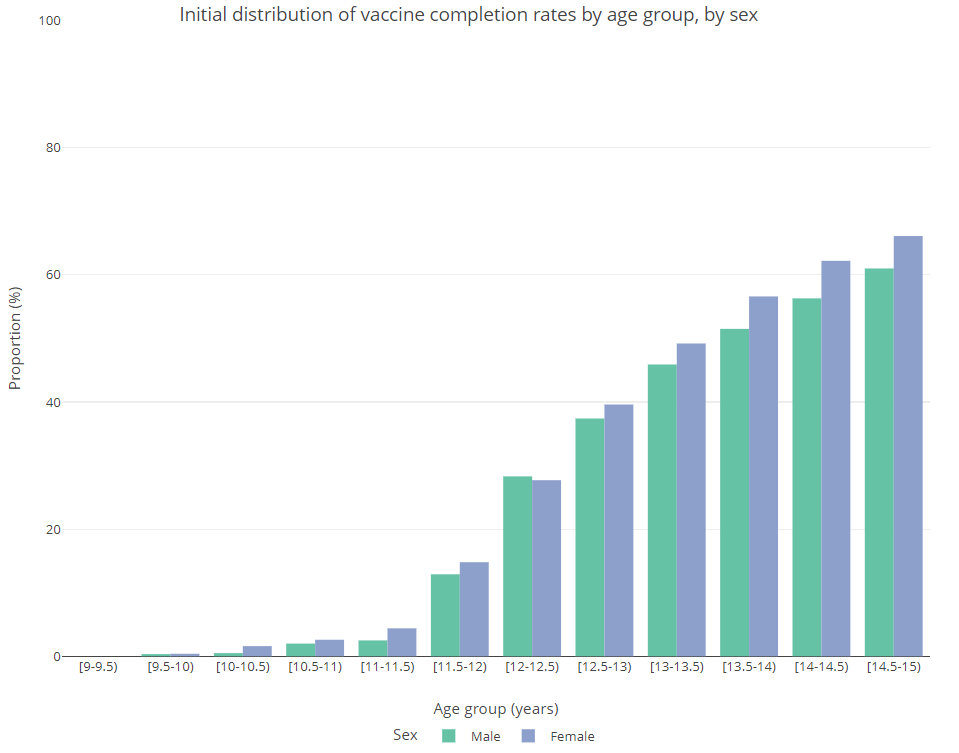


Figure S8: Initial distribution of vaccine completion rates in 2023 by age group, and compared by sex


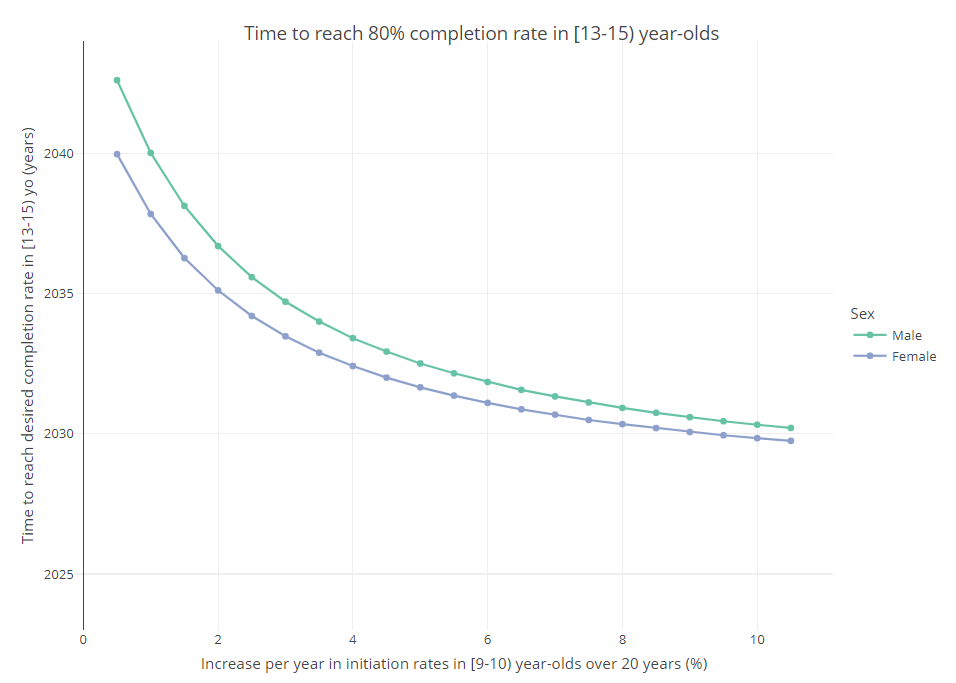


Figure S9: Time to reach a desired completion rate in [13-15) year-olds, by sex, as a function of the total annual increase (baseline annual increase + additional increase) in the initiation rates/probabilities in 9- and 9.5-year-olds

## Stratification by income

Figure S10 shows the initial distribution of vaccine completion rates in 2023 by age group, and compared by income. Completion rates are generally highest among those below poverty, and lowest for those with “unknown” income. Figure S11 shows how the time to reach a desired completion rate in [13-15) year-olds changes, by income. For a given increase in proactive initiation rates, the time it takes for those below poverty to reach the desired completion rates is shorter compared to those above poverty and with “unknown” income. As the initiation rate per year increases, differences across income become less pronounced. This suggests that interventions that substantially increase initiation rates in 9-year-olds lead to reductions that are differential across income levels.


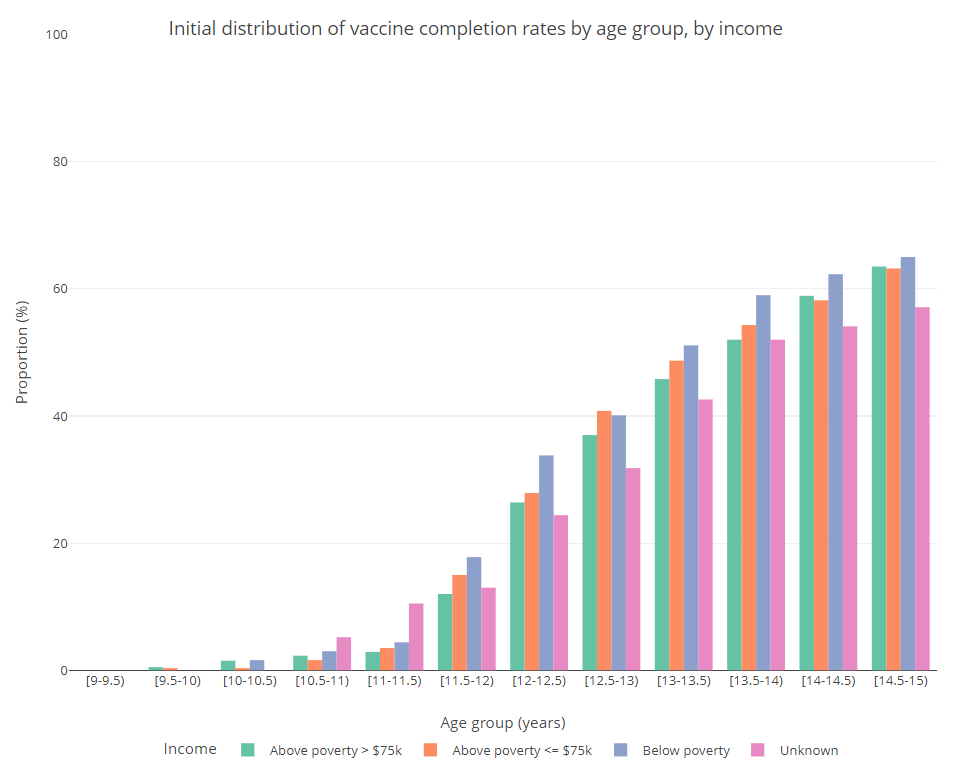


Figure S10: Initial distribution of vaccine completion rates in 2023 by age group, and compared by income


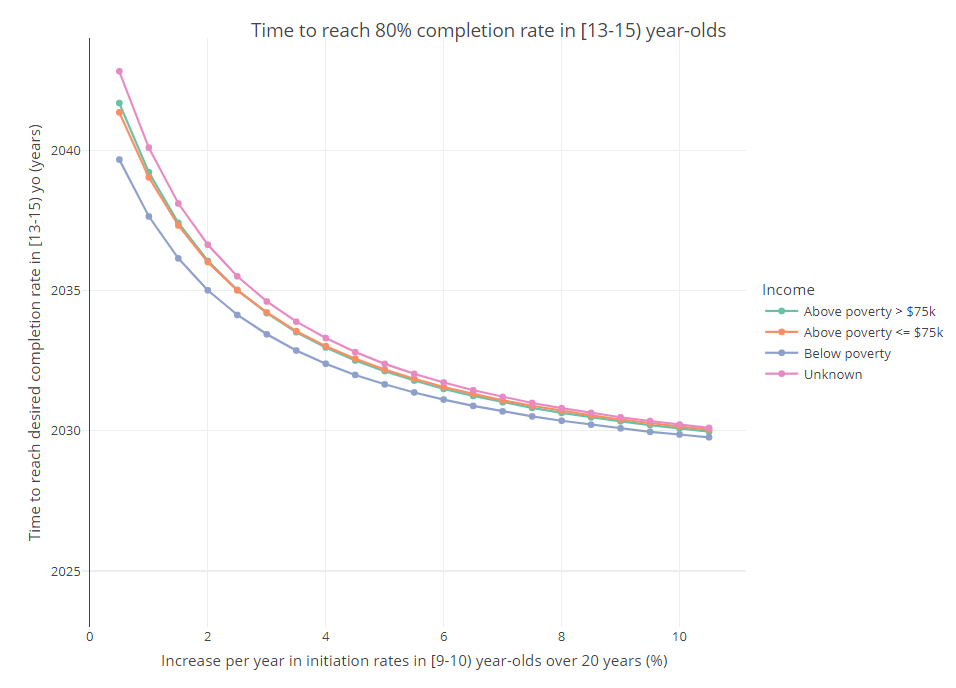


*Figure S11: Time to reach a desired completion rate in [13-15) year-olds, by income, as a function of the total annual increase (baseline annual increase + additional increase) in the initiation rates/probabilities in 9- and 9.5-year-olds*

## Stratification by census region

Figure S12 shows the initial distribution of vaccine completion rates in 2023 by age group, and compared by census region. Completion rates are generally higher among those that live in the Northeast and Midwest regions, and lowest for those in the South region. Figure S13 shows that, for a given increase in proactive initiation rates, the time it takes for those that live in the Northeast to reach the desired completion rates is shorter compared to those that live in the South and West regions. Again, as the initiation rate per year increases, differences across regions become less pronounced.


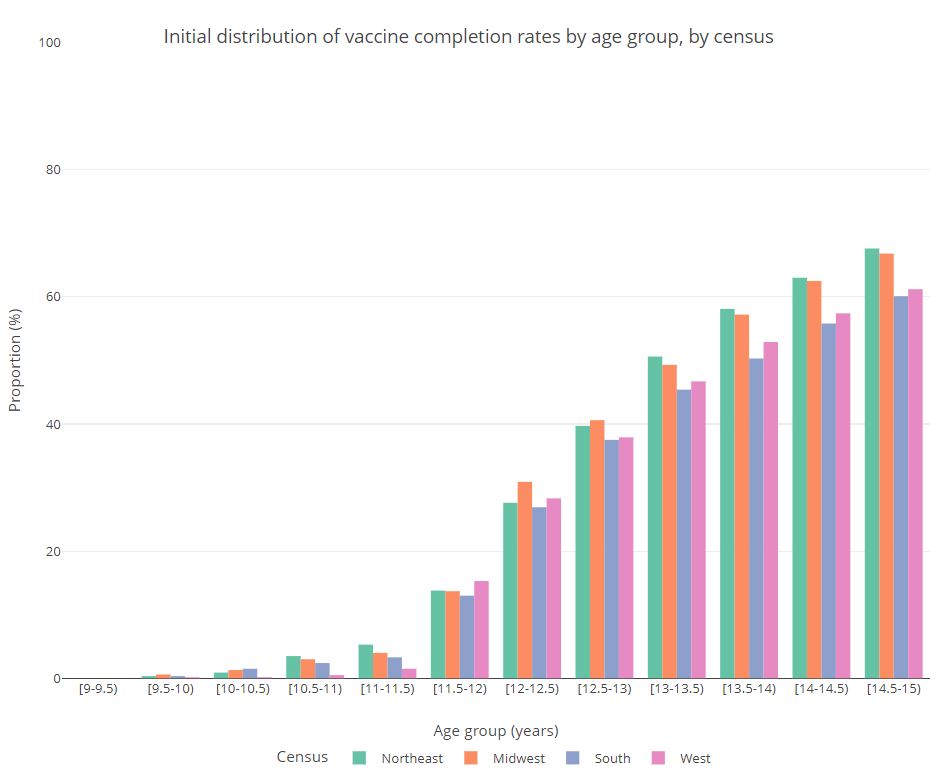


Figure S12: Initial distribution of vaccine completion rates in 2023 by age group, and compared by census region


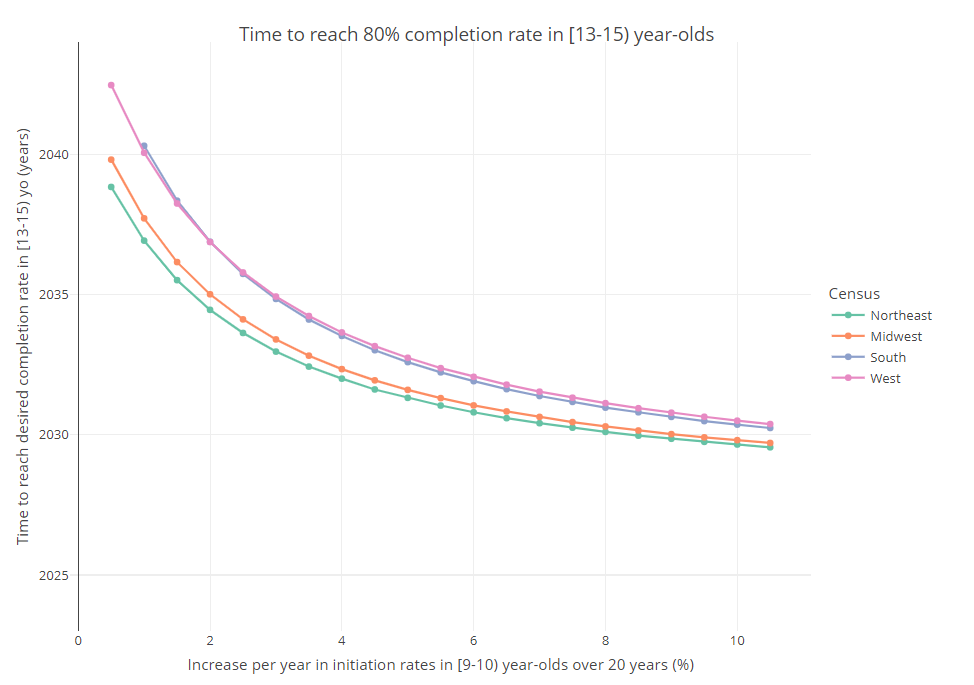


*Figure S13: Time to reach a desired completion rate in [13-15) year-olds, by census region, as a function of the total annual increase (baseline annual increase + additional increase) in the initiation rates/probabilities in 9- and 9.5-year-olds*

## Stratification by facility

Figure S14 shows the initial distribution of vaccine completion rates in 2023 compared by vaccine administration facility. In general, completion rates are highest in private facilities and lowest in STD/school/teen clinics, compared with other known facility types. Figure S15 shows that the time it takes for those that received their vaccines in private facilities or in “mixed” facilities to reach the desired completion rates is shorter (by up to 7 years when the total annual increase in proactive vaccination rates is 2.5%) compared to those that receive their vaccination in STD/school/teen clinics. As the initiation rate increase per year increases, differences across facility type become less pronounced.


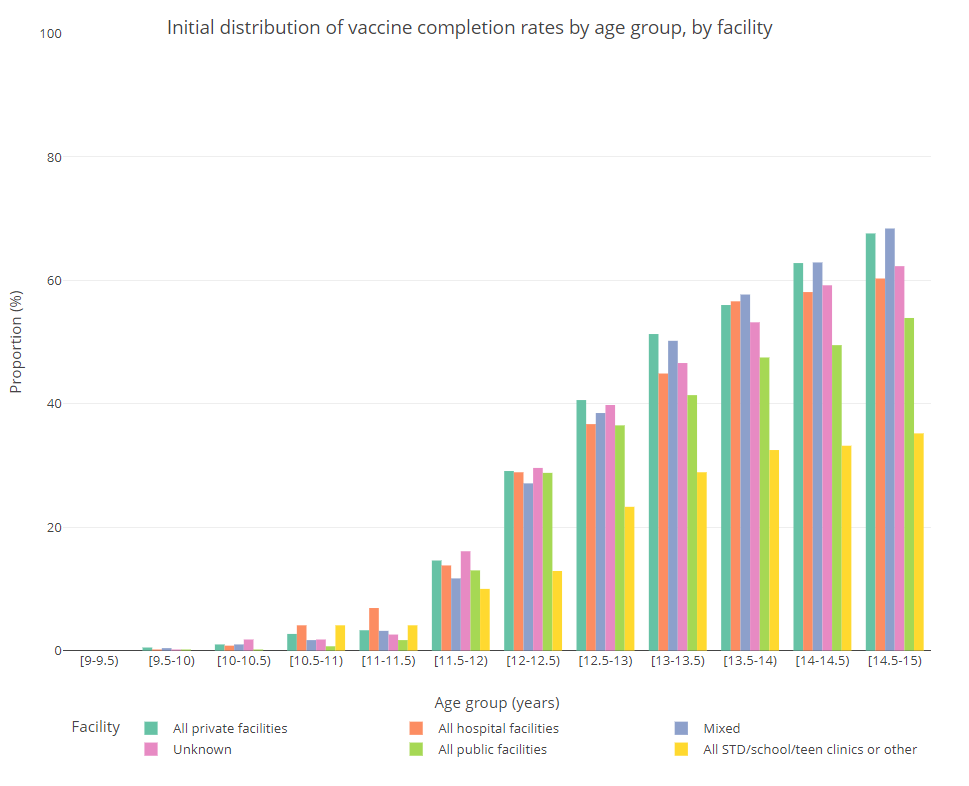


Figure S14: Initial distribution of vaccine completion rates in 2023 by age group, and compared by facility.


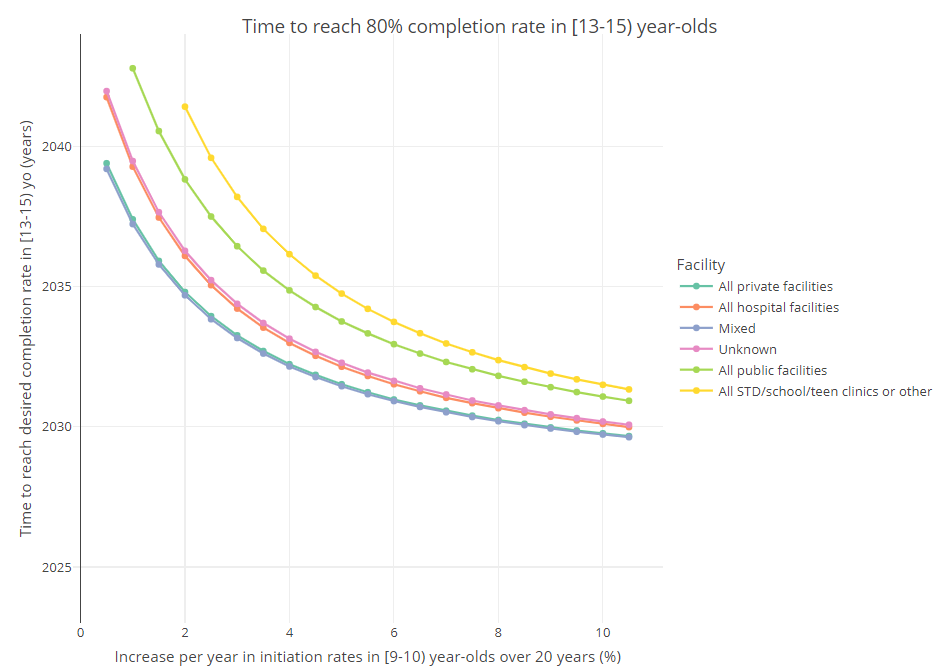


Figure S15: Time to reach a desired completion rate in [13-15) year-olds, by facility, as a function of the total annual increase (baseline annual increase + additional increase) in the initiation rates/probabilities in 9- and 9.5-year-olds

# References

1. https://www.cdc.gov/mmwr/volumes/71/wr/mm7135a1.htm#T1_down [↑](#endnote-ref-1)
2. <https://www.cdc.gov/vaccines/imz-managers/nis/datasets-teen.html> [↑](#endnote-ref-2)
3. Meites, E., Kempe, A., & Markowitz, L. E. (2016). Use of a 2-dose schedule for human papillomavirus vaccination—updated recommendations of the Advisory Committee on Immunization Practices. *Morbidity and Mortality Weekly Report*, *65*(49), 1405-1408. [↑](#endnote-ref-3)
4. Respondents who received their initiation and completion or second and third dose in the same 6-month age group are censored from this derivation. [↑](#endnote-ref-4)
5. The main issue can be illustrated considering two random teens taken from the dataset who reported an age of 10 years for when they got their 1^st^ HPV dose. When both teens have different ages, then their 1^st^ dose must have been administered at different calendar years, so they are in principle not comparable. Alternatively, when they have the same age (i.e., they got their 1^st^ dose during the same calendar year), there are 5 calendar years in which the dose administration could have happened (in this case, 2013-2017) because the survey consists of teens aged 13 to 17. [↑](#endnote-ref-5)
6. Assumed. [↑](#endnote-ref-6)
